# Extracellular vesicles from adipose-derived stem cells ameliorate ultraviolet B-induced skin photoaging by attenuating reactive oxygen species production and inflammation

**DOI:** 10.1186/s13287-020-01777-6

**Published:** 2020-07-01

**Authors:** Peng Xu, Yu Xin, Zheng Zhang, Xiangyu Zou, Ke Xue, Huizhong Zhang, Wenjie Zhang, Kai Liu

**Affiliations:** 1 Department of Plastic and Reconstructive Surgery, Shanghai Key Laboratory of Tissue Engineering, Shanghai Ninth People’s Hospital, Shanghai Jiao Tong University School of Medicine, Shanghai, 200011 China; 2 Department of Urology, Shanghai Children’s Medical Center, Shanghai Jiao Tong University School of Medicine, Shanghai, 200127 China

**Keywords:** Extracellular vesicles, Adipose-derived stem cells, Photoaging, ROS, Inflammation

## Abstract

**Background:**

Large numbers of adipose-derived stem cells (ADSCs) are easily obtained and have been demonstrated to protect against ultraviolet B (UVB)-induced skin photoaging. Extracellular vesicles (EVs) exhibit some of the same effects as the cells from which they originate and have many advantages over stem cells. In particular, their application circumvents many safety concerns associated with cell therapy. Thus, as a cell-free agent, adipose-derived stem cell extracellular vesicles (ADSC-EVs) have anti-photoaging potential. However, the protective effects of ADSC-EVs in skin photoaging remain uncertain.

**Methods:**

To investigate the effect of ADSC-EVs on mice with UVB-induced photoaging, 150 μg and 300 μg ADSC-EVs were subcutaneously injected weekly into photoaging mice for 8  weeks. The protective effect was evaluated by gross assessment and hematoxylin and eosin, Masson’s trichrome, and β-galactosidase staining. Proliferating cell nuclear antigen, CD68, and dihydroethidium staining were performed to evaluate cell proliferation, inflammation infiltration, and reactive oxygen species (ROS) production, respectively. In vitro, 100 μg/mL and 200 μg/mL ADSC-EVs were used to treat photoaging fibroblasts (FBs). β-galactosidase staining and collagen 1 and matrix metalloproteinase 3 (MMP-3) expression were analyzed to evaluate FB senescence. To explain the protective mechanism of ADSC-EVs, their role in regulating ROS production, antioxidant enzyme expression, cell cycle arrest, and inflammation was evaluated.

**Results:**

In vivo, we showed that ADSC-EVs decreased skin wrinkles in mice with UVB-induced photoaging, while promoting epidermal cell proliferation and attenuating macrophage infiltration and ROS production. In vitro, we showed that ADSC-EVs increased FB activity and protected FBs from UVB-induced senescence, attenuated raw 264.7 cell differentiation from M0 to M1 macrophages, reduced intracellular ROS production, promoted antioxidant enzyme expression, and rescued FBs from cell cycle arrest.

**Conclusion:**

The anti-photoaging effect of ADSC-EVs was attributed to their ability to attenuate ROS production and the inflammatory response, which are key factors in MMP activation and collagen degradation.

**Graphical abstract:**

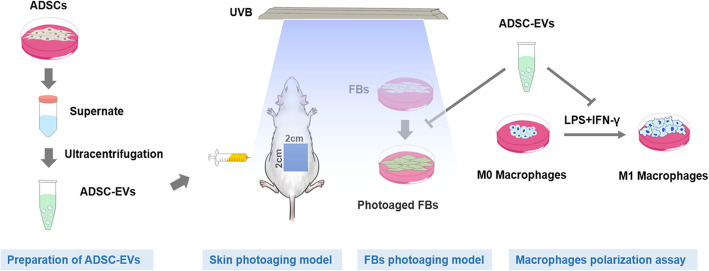

## Background

Skin photoaging is characterized by wrinkles, loss of elasticity, dryness, laxity, and rough texture [1]. Furthermore, it has a tendency to develop into skin tumors [2]. Researchers have therefore been exploring methods to slow photoaging. The topical application of antioxidants and growth factors has proven to be effective in preventing photoaging. However, their usage has been limited by poor permeability and stability [2–4]. Adipose-derived stem cells (ADSCs) have been demonstrated to protect against ultraviolet B-induced skin photoaging [5–7], though there are concerns regarding tumorigenicity and risk of contamination from their production by cell culture [8]. Recent studies have demonstrated that the therapeutic effects of stem cells can be mainly attributed to paracrine mechanisms rather than their differentiation [9].

Among the paracrine products of stem cells, extracellular vesicles (EVs) play an important role. EVs exhibit some of the same effects as the cells from which they originate and have many advantages over stem cells as therapeutic agents, such as they are more stable, stored more easily, have no risk of aneuploidy, and have a lower possibility of immune rejection following in vivo allogeneic administration [10]. Thus, adipose-derived stem cell extracellular vesicles (ADSC-EVs) as an “off-the-shelf” bioactive material, have the potential to carry out anti-photoaging, given that ADSCs have been proven to conduct this process efficiently [5–7]. More importantly, ADSC-EVs are especially suitable for clinical application as they can be easily obtained as a byproduct of liposuction on a large scale [11].

At present, the protective effects of ADSC-EVs in skin photoaging remains uncertain. To this end, ADSC-EVs were injected subcutaneously into nude mice to investigate their effects in vivo. The role of ADSC-derived EVs in regulating reactive oxygen species (ROS) production and inflammation, two key mechanisms underlying skin photoaging, was evaluated in vitro.

## Methods

### Cell isolation and culture

As described previously, human ADSCs were isolated from fat granules discarded from patients who underwent liposuction [12]. After isolation, ADSCs were cultured in low-glucose Dulbecco’s modified Eagle’s medium (DMEM, Invitrogen, Thermo Fisher Scientific, Inc.) supplemented with 10% fetal bovine serum (FBS, HyClone, GE Healthcare Life Sciences, Logan, UT, USA). Cells were maintained at 37 °C in a humidified atmosphere containing 5% CO_2_ and passaged every 3–5 days. Cells from passage 3 were used for EV preparation. ADSC (passage 3) characterization was performed by inducing adipogenic, osteogenic, and chondrogenic differentiation combined with surface marker analysis.

Human FBs were isolated from foreskin specimens discarded from circumcisions, as previously described [13]. FBs were cultured in high-glucose DMEM (Invitrogen, Thermo Fisher Scientific, Inc.) supplemented with 10% FBS. Cells were maintained at 37 °C in a humidified atmosphere containing 5% CO_2_ and passaged every 3–4 days. Cells from passages 3–5 were used in experiments. Informed consent was obtained from all patients. The study protocol was approved by the Ethics Committee of Shanghai Ninth People’s Hospital.

To investigate the role of EVs in regulating inflammation in vitro, mouse macrophage raw 264.7 cells were purchased from the ATCC cell bank. They were cultured in high-glucose DMEM supplemented with 10% FBS and maintained at 37 °C in a humidified atmosphere containing 5% CO_2_.

### In vitro differentiation of ADSCs

The trilineage differentiation of ADSCs was performed as previously described [14]. All chemicals were purchased from Sigma (St. Louis, MO, USA) unless otherwise stated. For adipogenic differentiation, ADSCs were cultured in the adipogenic induction medium (DMEM containing 10% FBS, 10 μg/mL insulin, 10^− 6^ M dexamethasone, and 100 μg/mL 3-isobutyl-methylxanthine) for 21 days, followed by Oil Red O staining. For osteogenic differentiation, ADSCs were cultured in the osteogenic induction medium (DMEM containing 10% FBS, 10^− 7^ M dexamethasone, 10 mM/L β-glycerophosphate, and 50 μg/mL ascorbic acid) for 21 days, followed by Alizarin Red staining. For chondrogenic differentiation, 5 × 10^5^ ADSCs were pelleted by centrifugation in a 15-mL culture tube and then cultured in the chondrogenic induction medium (Chondro BulletKit, Lonza Walkersville, Walkersville, MD, USA). After 21 days, the pellet was fixed in 4% paraformaldehyde, embedded in paraffin, sectioned, and stained with Alcian Blue solution.

### Preparation of EVs

After reaching 80% confluency, passage 3 ADSCs were rinsed with phosphate-buffered saline (PBS) and cultured with serum-free medium for 48 h. The conditioned medium was collected and centrifuged to isolate and purify EVs following a previously constructed protocol [15, 16]. Briefly, the conditioned medium was centrifuged at 3000×*g* for 30 min at 4 °C, followed by filtering with a 0.45-μm and 0.22-μm filter (SteritopTM, Millipore, USA) to remove the remaining cells and cellular debris. Finally, EVs were isolated by size fractionation and concentrated by centrifugation using an ultra-clear tube (Millipore) with a molecular weight cutoff of 100 kDa. EVs were stored at − 80 °C for the following experiments. Nanoparticle tracking analysis (NTA, Zeta View PMX 110, Particle Metrix, Meerbusch, Germany), transmission electron microscopy (TEM, JEOL microscope, JSM-7001TA, Tokyo, Japan), and western blot were used to identify EVs.

### Cellular uptake of EVs

As previously described [17], EVs were labeled by CM-Dil red fluorescent membrane linker dye (C7000, Invitrogen, Waltham, MA, USA) according to the manufacturer’s instructions. Briefly, 200 μg EVs suspended in 500 μL PBS were labeled by 5 μL CM-Dil stock solution (1 mg/mL) and were incubated at 37 °C for 5 min and 4 °C for 15 min. After incubation, EVs were repeatedly washed with PBS via ultrafiltration centrifugation to remove unbound CM-Dil. FBs were incubated with CM-Dil-labeled EVs (100 μg/mL) for 12 h. Then, FBs were washed three times with PBS, fixed in 4% paraformaldehyde, and stained with phalloidin and DAPI. Finally, cells were observed under a Zeiss Confocal LSM 710 microscope (Carl Zeiss, Jena, Germany) to determine the uptake of the labeled EVs.

### UVB-induced skin photoaging model and treatment

All animal experiments complied with the National Institutes of Health Guide for the Care and Use of Laboratory Animals. The procedures were approved by the Animal Research Committee of Shanghai Jiao Tong University Affiliated Ninth People’s Hospital. A total of 40 female BALB/c nude mice (5 weeks old) were purchased from Shanghai Chuansha Experimental Animal Raising Farm (Shanghai, China) and raised in a specific pathogen-free environment for 1 week to adapt to the new environment. Then, mice were randomly assigned to four groups (*n* = 10): [1] Control group: no UVB irradiation and treatment [2]; UVB group: UVB irradiation + subcutaneous injection of 1 mL PBS/week [3]; UVB + 150 μg/week group: UVB irradiation + subcutaneous injection of EVs (150 μg in 1 mL solution/week); and [4] UVB + 300 μg/week group: UVB irradiation + subcutaneous injection of EVs (300 μg in 1 mL solution/week). EVs were suspended in PBS and injected at the same dorsal subcutaneous position (2 cm × 2 cm, indicated in the graphic abstract) for each nude mouse. The applied dose of EVs was determined according to previous report [18] and preliminary experiment.

The UVB irradiation procedure was conducted as previously described [12]. In detail, nude mice were irradiated under a UVB lamp (Philip, 311 nm, 20 W/01, Germany) for 8 weeks, five times a week. The distance between the dorsum of the animals and the lamps was 9 cm. The energy density was measured with a UVB energy detector (UV-DETECTOR 150, Ergu, China). The irradiation dose was one minimal erythema dose (MED) of 160 mJ/cm^2^ in the first week, followed by 210 mJ/cm^2^, 280 mJ/cm^2^, and 370 mJ/cm^2^ in weeks 2 to 4, and 370 mJ/cm^2^ in weeks 5 to 8. The total UVB dose was approximately 80 MED (12.7 J/cm^2^).

### Gross assessment of skin photoaging

After UVB irradiation and EV injection, nude mice were anesthetized with a rodent anesthesia machine (Medical Supplies & Services INT. LTD., Keighley, UK). The skin wrinkles, skin roughness, and dermal thickness (though polarized light mode) were recorded by a multifunctional skin detector (CBS-802, Dermoscopy Skin Analyzer, China) according to the manufacturer’s instructions. The three-dimensional roughness of the skin was produced by the detector software. A classical clinical wrinkle score system was used to evaluate the general photoaging of the skin, as previously described [19]. A higher score indicated more severe skin photoaging.

### Histological analyses

Skin specimens were taken from the injection area of the dorsum of each mouse, fixed in 4% paraformaldehyde, paraffin-embedded, and sectioned at 5 μm for hematoxylin and eosin (H&E) and Masson’s trichrome staining. Five randomly selected fields from each specimen were imaged with light microscopy (Nikon Eclipse 90i, Japan), and the epidermal and dermal thickness of each sample were measured using Image-Pro Plus 6 software (Rockville, MD, USA), as previously described [12].

### Immunohistochemical and immunofluorescence staining

For specimen immunohistochemical and immunofluorescence staining, anti-proliferating cell nuclear antigen (PCNA) (1:200), anti-CD68 (1:300) (both from Abcam, Cambridge, UK), and anti-β galactosidase (1:50; Proteintech, Rosemont, USA) antibodies were used as previously described [20, 21]. Briefly, sections were dewaxed and hydrated followed by antigen retrieval. The endogenous peroxidase activity was quenched by immersion in 2% (v/v) hydrogen peroxide for 5 min. After washing in PBS, the sections were blocked with 5% goat serum (Beyotime, China) for 30 min at room temperature, followed by incubation with the primary antibody overnight at 4 °C. HRP-labeled (Dako, Glostrup, Denmark) or PE-labeled (Jackson ImmunoResearch, Carlsbad, CA, USA) secondary antibodies were separately added for 30 min at 37 °C. A 3,3′-diaminobenzidine solution (DAB Substrate Kit, Burlingame, CA, USA) was used to visualize the immunohistochemical reaction, followed by counterstaining with hematoxylin. DAPI (1:1000; Boster, Wuhan, China) was used to stain nuclei in immunofluorescence staining. Five randomly selected fields from each specimen were imaged with light microscopy (Nikon Eclipse 90i, Japan) or fluorescence microscopy (Olympus, Tokyo, Japan). The number of PCNA-, CD68-, and β-galactosidase-positive cells was calculated using Image-Pro Plus 6 software (Rockville, MD, USA). The epidermal or dermis proliferate index was calculated as being equal to the number of epidermal or dermis PCNA-positive cells/the number of total epidermal or dermis cells × 100%.

For cell immunofluorescence staining, cells in 24-well plates were fixed in 4% paraformaldehyde for 10 min. After washing in PBS, holes were punched in cells by 0.25% (v/v) Triton for 10 min. After washing once again with PBS, cells were blocked with 5% goat serum (Beyotime, China) for 30 min at room temperature, followed by incubation with PE-conjunct anti-iNOS (1:500, Biolegend, San Diego, CA, USA) overnight at 4 °C. Cells were then counterstained with DAPI (1:1000, Boster, Wuhan, China) and examined using a fluorescence microscope (Olympus Corporation, Tokyo, Japan). Five randomly selected fields from each well were imaged (*n* = 3), and the number of iNOS-positive cells was calculated using Image-Pro Plus 6 software (Rockville, MD, USA).

### In vitro UVB irradiation of human dermal fibroblasts

Human fibroblasts were cultured in 96-well (2000 cells/well) or 6-well plates (1 × 10^5^ cells/well) in a culture medium, supplemented with or without different doses of EVs, at 37 °C for 24 h. After the supernatant was removed, cells were washed with PBS twice and covered by a thin layer of PBS. Then, cells were exposed to UVB light (Philip, 311 nm, 20 W/01, Germany) at a total dose of 100 mJ/cm^2^ as previously described [22]. After irradiation, PBS was removed and replaced with culture medium for 24 or 72 h for further experiments.

### Cell activity assays

Cell activity was measured with a Cell Counting Kit-8 (CCK8, Dojindo Laboratories, Kumamoto, Japan) at 72 h after UVB irradiation according to the manufacturer’s instructions. After incubation with the CCK-8 kit for 2 h, the absorbance value was measured with a microplate reader (Thermo Electron Corporation, USA) at a wavelength of 450 nm. Results are expressed as follows:

$$ \mathrm{Total}\ \mathrm{cellular}\ \mathrm{activity}\ \left(\%\mathrm{of}\ \mathrm{control}\right)=\frac{\mathrm{Absorbance}\ \mathrm{of}\ \mathrm{treatment}\ \mathrm{group}}{\mathrm{Absorbance}\ \mathrm{of}\ \mathrm{control}\ \mathrm{group}}\times 100\% $$

The EV concentrations of the following experiments were determined by cell activity assays with and without UVB irradiation.

### Cell β-galactosidase (SA-β-Gal) staining

Cell SA-β-Gal staining was performed using a senescence β-galactosidase staining kit (C0602, Beyotime, China) at 72 h after UVB irradiation according to the manufacturer’s instructions. SA-β-Gal-positive cells were imaged with an inverted microscope (Carl Zeiss, Oberkochen, Germany) and counted using Image-Pro Plus 6.0 software (Rockville, MD, USA). Five randomly selected fields from each sample (*n* = 3 samples/group) were selected to determine the rate of cell aging:

$$ \frac{Number\ of\ \mathrm{SA}-\upbeta -\mathrm{Gal}-\mathrm{positive}\ \mathrm{cell}\mathrm{s}}{\mathrm{Total}\ \mathrm{cell}\ \mathrm{number}}\times 100\% $$

### In vitro polarization induction of raw 264.7 macrophages and treatment

To investigate the role of ADSC-EVs in regulating macrophage polarization in an inflammatory environment, 5 × 10^5^ raw 264.7 cells were seeded in 6-well plates. After cell attachment, the culture medium was replaced with culture medium (control group), culture medium containing 1 mg/mL LPS (Sigma-Aldrich, St. Louis, MO, USA) and 20 ng/mL IFN-γ (PeproTech, Rocky Hill, USA) (LPS + IFN-γ group), or culture medium containing 1 mg/mL LPS, 20 ng/mL IFN-γ, and different concentrations of EVs (LPS + IFN-γ + EV group), as previously described [23]. After incubating the different media for 24 h, the polarization of raw 264.7 cells to M1/M2 macrophages was identified by flow cytometry, immunofluorescence staining, and quantitative real-time polymerase chain reaction (qPCR).

### Flow cytometry for cell surface marker detection

Cells were suspended in flow cytometry staining buffer (Invitrogen, San Diego, CA, USA) and filtered into a single-cell suspension with a 40 μm/100 μm mesh. Then, 5 × 10^5^ cells were incubated with FITC-anti-mouse CD86 (1:50), PE-anti-mouse CD206 (1:40), PE-anti-human CD19 (1:40), PE-anti-human CD34 (1:40), PE-anti-human CD11b (1:40), PE-anti-human CD45 (1:40), FITC-anti-human HLA-DR (1:50), PE-anti-human CD73 (1:40), FITC-anti-human CD90 (1:40), and PE-anti-human CD105 (1:40) antibodies (all from BioLegend, San Diego, CA, USA) at 4 °C for 30 min. After washing twice with staining buffer, cells were suspended in 100 μL staining buffer and analyzed via flow cytometry (BD FACS Calibur, Beckman Coulter).

### Detection of ROS

ROS levels were detected using in situ dihydroethidium red fluorescence staining kit (S0063, Beyotime, China) according to the manufacturer’s instructions. Briefly, the skin tissues were embedded in optimal cutting temperature compound and cut into 8-μm cryosections. After washing with PBS, sections were incubated with dihydroethidium solution (5 μmol/L) at 37 °C for 30 min. Nuclei were stained with DAPI (1:1000; Boster, Wuhan, China). Five randomly selected fields from each section were imaged with a fluorescence microscope (Olympus, Tokyo, Japan). The mean fluorescence intensity of dihydroethidium-positive cells was calculated using Image-Pro Plus 6 software (Rockville, MD, USA).

Intracellular ROS levels were detected using an ROS assay kit (S0033, Beyotime, China) according to the manufacturer’s instructions. Briefly, FBs were incubated with DCFH_2_-DA (10 μM) at 37 °C for 20 min. After washing three times with DMEM, FBs were exposed to UVB irradiation. Then, half of the FBs were observed and imaged under a fluorescence microscope (Olympus Corporation, Tokyo, Japan) at an excitation wavelength of 488 nm. The other half of the FBs were collected and quantitatively analyzed by flow cytometry (BD FACS Calibur, Beckman Coulter).

### Cell cycle analysis

Cell cycle analysis was performed using a cell cycle and apoptosis analysis kit (C1052, Beyotime, China) at 24 h after UVB irradiation, according to the manufacturer’s instructions. Briefly, 5 × 10^5^ cells were harvested and fixed in 70% ethanol at 4 °C overnight. After washing twice with PBS, cells were incubated with staining solution (a mixture containing 25 μL 20× propidium iodide, 10 μL 50× RNase A, and 0.5 mL staining buffer) at 37 °C for 30 min in the dark. DNA content was determined by flow cytometry (BD FACS Calibur, Beckman Coulter).

### Quantitative real-time polymerase chain reaction

At 48 h after UVB irradiation and 24 h after inducing polarization of raw 264.7 macrophages, total cellular mRNA was extracted using Trizol reagent (Invitrogen, Carlsbad, CA) according to the manufacturer’s instructions. Reverse transcription for cDNA synthesis was then performed using a reverse transcription master mix (EZBioscience, Roseville, USA). qPCR was performed using a SYBR green qPCR master mix (ROX2 plus) (EZBioscience, Roseville, USA). A hot start at 95 °C for 5 min was followed by 40 cycles at 95 °C for 10 s and at 60 °C for 30 s. β-actin was used for normalization. Normalized expression levels were calculated using the 2^-ΔΔCT^ method and are presented as fold increases relative to the negative control. The primers for qRT-PCR are listed in Table 1.

**Table 1:** Primers used for real-time qPCR

Genes	Sequences (5′ to 3′)	
Human Col 1	F	AGG GCC AAG ACG AAG ACA TC
	R	GTC GGT GGG TGA CTC TGA GC
Human MMP3	F	TGA CAC ACA CTT TGA AGA GTA AC
	R	TCA CAG AGA CTT AGG TGA AGA AT
Human SOD 1	F	GGG AAG CAT TAA AGG ACT GA
	R	CAC CGT GTT TTC TGG ATA GAG
Human CAT	F	CAA CAC TGC CAA TGA TGA TA
	R	GTT CTT GAC CGC TTT CTT CT
Human β-actin	F	AAG GTG ACA GCA GTC GGT T
	R	TGT GTG GAC TTG GGA GAG G
Mouse TNF-α	F	CCTGTAGCCCACGTCGTAG
	R	GGGAGTAGACAAGGTACAACCC
Mouse IL-6	F	TAGTCCTTCCTACCCCAATTTCC
	R	TTGGTCCTTAGCCACTCCTTC
Mouse β-actin	F	GTGACGTTGACATCCGTAAAGA
	R	GCCGGACTCATCGTACTCC

### Western blotting

At 72 h after UVB irradiation and 24 h after inducing polarization of raw 264.7 macrophages, cells were harvested and lysed with RIPA (Beyotime, China) buffer containing 1 mM phenylmethanesulfonyl fluoride (PMSF, Beyotime, China). Protein concentrations were determined using a BCA Protein Assay Kit (Beyotime, China). For each sample, 30 μg total protein was loaded onto a 10–15% SDS-PAGE gel. Proteins were transferred to a PVDF membrane (Millipore) and incubated with primary anti-CD9, anti-CD63, anti-CD81, anti-TSG101, anti-GM130 (all from Abcam, Cambridge, UK, 1:1000), anti-collagen 1 (Col-1), anti-matrix metalloproteinase 3 (MMP-3), anti-superoxide dismutase 1 (SOD-1), anti-catalase (CAT), anti-P53, anti-P21, anti-P50, anti-p-P50, and anti-β-actin (all from CST, Danvers, MA, USA, 1:1000) antibodies. Protein expression was visualized and normalized against β-actin.

### Statistical analysis

Numerical data are presented as the mean ± standard deviation and were analyzed by one-way analysis of variance (ANOVA), followed by Tukey’s post hoc test. Statistical analysis was performed using SAS (SAS Institute Inc., USA). *P* < 0.05 was considered to indicate a significant difference.

## Results

### Characterization of ADSCs and ADSC-EVs

ADSCs at passage 3 exhibited adipogenic, osteogenic, and chondrogenic differentiation capacity (Supplementary Fig. S1A-C). High expression levels of ADSC surface markers such as CD73 (97.01 ± 2.24%), CD90 (95.72 ± 2.13%), and CD105 (96.48 ± 1.83%) were observed, and almost no expression of negative markers such as CD19 (0.77 ± 0.24%), CD34 (1.00 ± 0.38%), CD11b (0.90 ± 0.35%), CD45 (0.83 ± 0.19%), and HLA-DR (1.18 ± 0.26%) was found (Supplementary Fig. S1D).

**Fig. 1:**
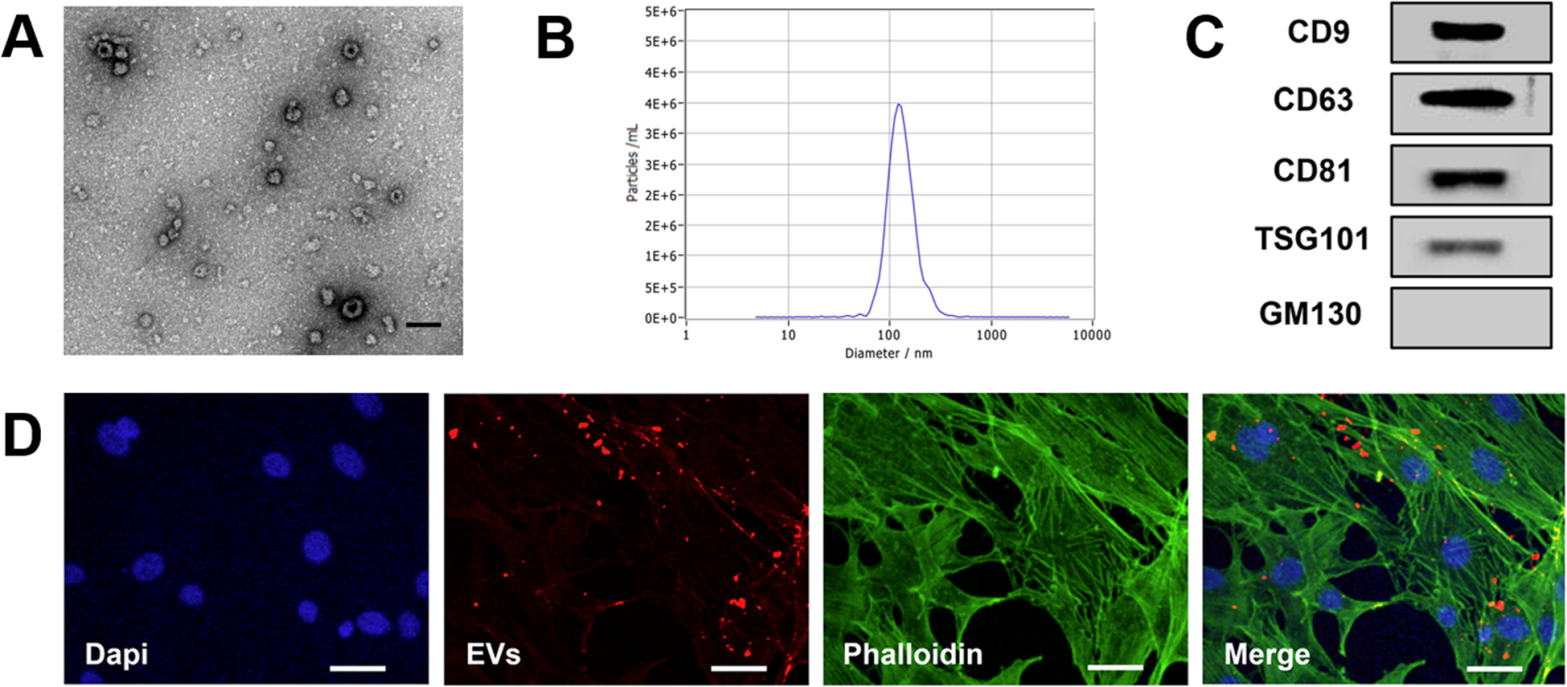
Characterization of adipose-derived stem cell extracellular vesicles (ADSC-EVs). **a** Round morphology shown by transmission electron microscopy (TEM). Scale bar = 200 nm. **b** Particle diameter distribution detected by nanoparticle tracking analysis (NTA), mean 127 ± 3 nm. **c** Expression of EV markers CD81, CD63, CD9, TSG101, and GM130, as detected by western blotting. **d** Immunofluorescence showing EVs internalized into fibroblasts after 12 h incubation. Scale bar = 50 μm

ADSC-EVs were round (Fig. 1a) with an average diameter of 127 ± 3 nm (Fig. 1b), confirmed by TEM and NTA, respectively. The positive expression of the EV markers CD9, CD63, CD81, and TSG101 and the negative expression of non-EV marker GM130 were confirmed by western blotting (Fig. 1c). After incubation with FBs for 12 h, EVs labeled with red fluorescence (CM-Dil) were observed to have been internalized into FBs (Fig. 1d). The concentration of 1 mg/mL EVs as determined by protein quantification was equivalent to 0.59 × 10^9^ particles/mL as determined by NTA.

### ADSC-EVs protected mice against UVB-induced skin photoaging

**Fig. 2:**
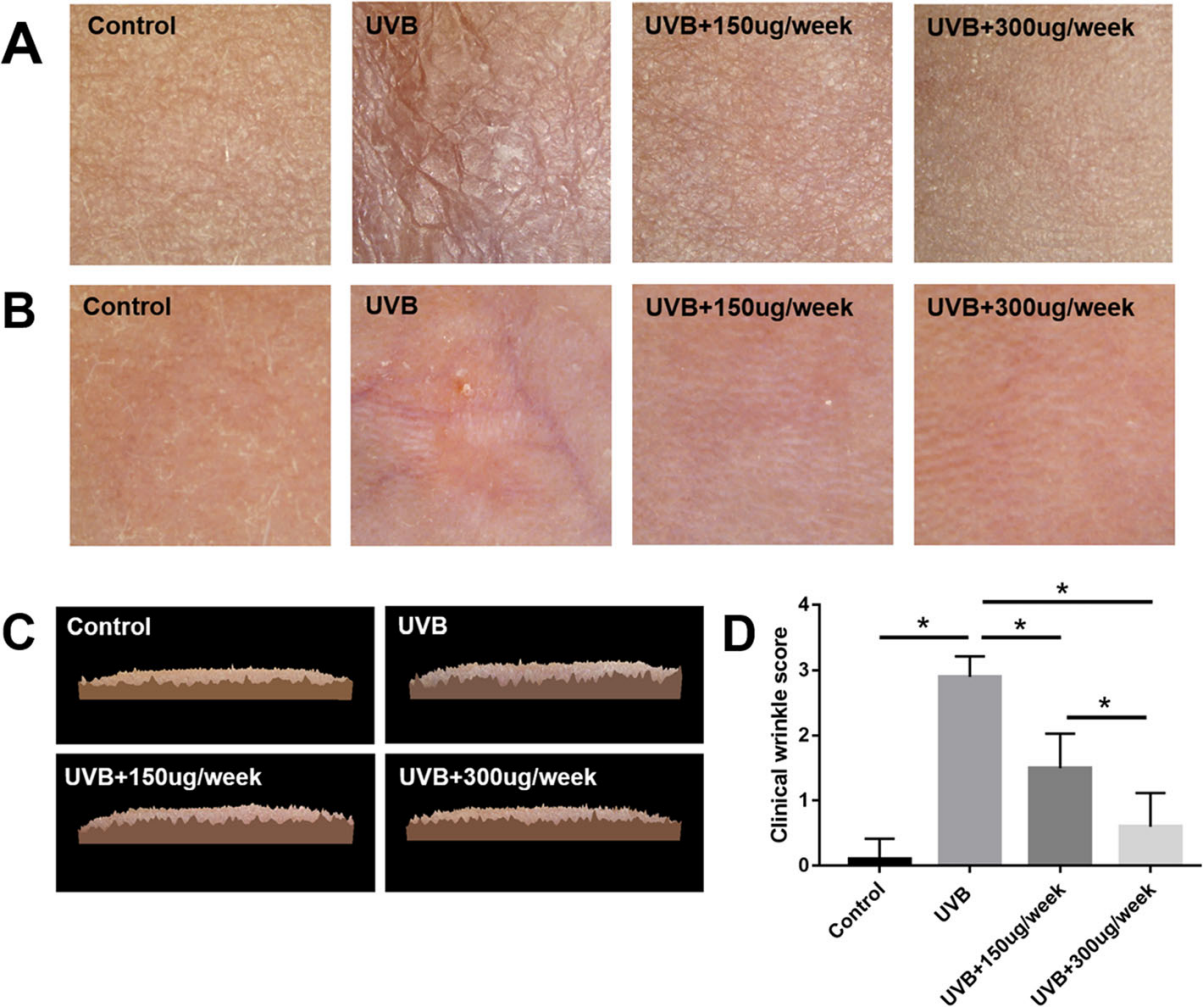
Skin photoaging. **a** Keratinization and wrinkles in the ultraviolet B (UVB) group and improvement in skin condition in the UVB + 150 μg/week and UVB + 300 μg/week groups. **b** Polarized light imaging skin thinning in the UVB group and improvement in the UVB + 150 μg/week and UVB + 300 μg/week groups. **c** Three-dimensional roughness was increased in the UVB group, which improved in the UVB + 150 μg/week and UVB + 300 μg/week groups. **d** Clinical wrinkle scores. *P < 0.05 indicated a significant difference

After 2 months of UVB exposure, the UVB group showed obvious skin keratinization and wrinkles relative to the control group (*P* < 0.05), as determined by gross observation and clinical wrinkle scoring. However, the skin texture in the UVB + 150 μg/week and UVB + 300 μg/week groups showed obvious dose-dependent improvement in skin condition (*P* < 0.05) (Fig. 2a, d). The three-dimensional roughness of the skin also was consistent, showing that EV injection reduced the degree of skin roughness (Fig. 2c). The polarized light mode showed UVB-induced skin thinning, which was reflected by the visibility of subcutaneous blood vessels. Meanwhile, the EV treatment groups (UVB + 150 μg/week and UVB + 300 μg/week groups) showed increased skin thickness relative to the UVB group (Fig. 2b).

**Fig. 3:**
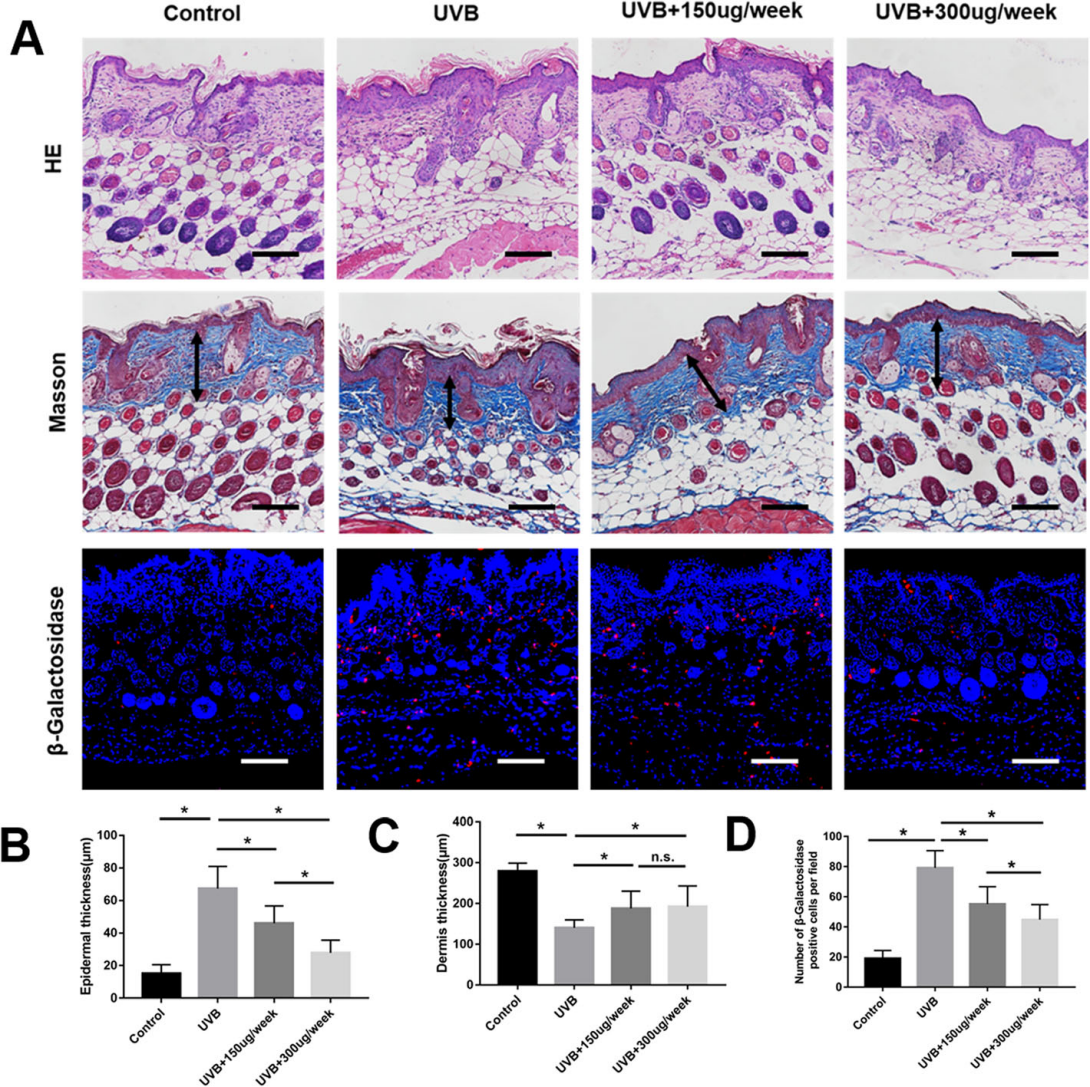
Histological assessment of photoaging. **a** Increased epidermal thickness, β-galactosidase expression, and decreased dermis thickness in the ultraviolet B (UVB) group. The appearance of skin photoaging improved in the UVB + 150 μg/week and UVB + 300 μg/week groups. Black arrows indicate dermis thickness. Scale bars = 150 μm. **b** Epidermal thickness statistics. **c** Dermis thickness statistics. **d** Skin β-galactosidase expression statistics. *P < 0.05 indicated a significant difference; n.s., no significant difference between groups

HE combined Masson stain showed that compared to the control group, the epidermal thickness was thicker and the dermis thickness was thinner in the UVB group (*P* < 0.05) (Fig. 3). The epidermal thickening was partly counteracted by EVs in a dose-dependent manner (*P* < 0.05) (Fig. 3b). The dermis thinning was also partly counteracted by 150 μg/week and 300 μg/week of EV injection, but there was no difference between UVB + 150 μg/week group and UVB + 300 μg/week group (*p >* 0.05) (Fig. 3c). To further evaluate the cell aging conditions, the skin tissue was stained with β-galactosidase. The results show that UVB significantly induced β-galactosidase expression in skin photoaging, and this effect was counteracted by EVs in a dose-dependent manner (*P* < 0.05) (Fig. 3a, d). Histologic evaluation thus indicates that ADSC-EVs significantly protected mice against UVB-induced skin photoaging.

### ADSC-EVs promote epidermal cell proliferation and attenuate macrophage infiltration with ROS production

**Fig. 4:**
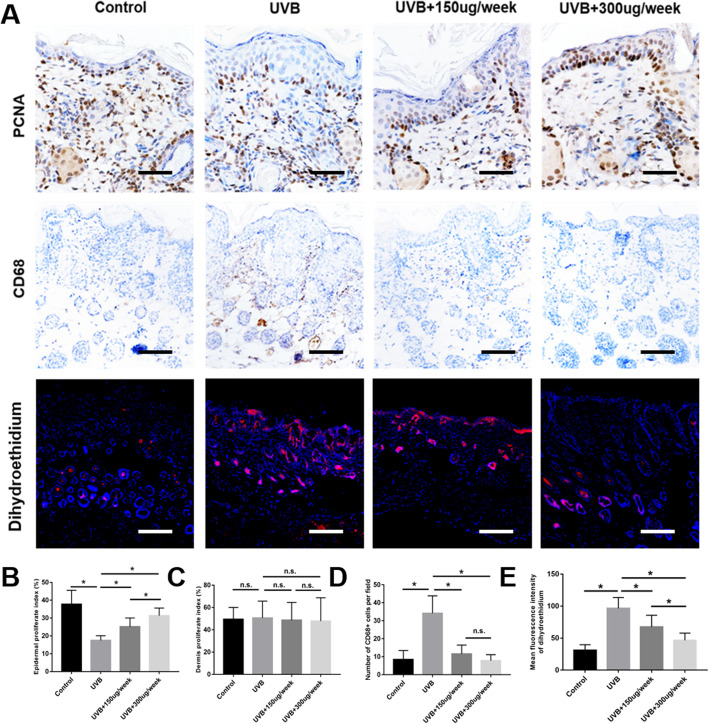
Cell proliferation, macrophage infiltration, and ROS production in photoaged skin. **a** Cell proliferation evaluated by proliferating cell nuclear antigen (PCNA) staining. Scale bars = 50 μm. Macrophage infiltration evaluated by CD68 staining. Scale bars = 100 μm. ROS production evaluated by dihydroethidium staining. Scale bars = 250 μm. **b** Epidermal proliferate indices. **c** Dermis proliferate indices. **d** Statistics for CD68-positive cell counts. **e** Skin ROS production statistics. *P < 0.05 indicated a significant difference; n.s., no significant difference between groups

Cell proliferation in the epidermal and dermis was measured by anti-PCNA immunohistochemical staining (Fig. 4a). No significant difference was observed in the dermis proliferation indices of the four groups (Fig. 4c). However, the epidermal proliferation indices in the UVB + 150 μg/week and UVB + 300 μg/week groups were significantly higher (*P* < 0.05) than those in the UVB group. In addition, the epidermal proliferation index in the UVB + 300 μg/week group was significantly higher (*P* < 0.05) than that in the UVB + 150 μg/week group (Fig. 4b).

Macrophage infiltration was measured by anti-CD68 immunohistochemical staining (Fig. 4a). Most CD68-positive cells were distributed in the dermis and subcutaneously. UVB exposure significantly increased numbers of CD68-positive cells (*P* < 0.05), whereas their numbers in the UVB + 150 μg/week and UVB + 300 μg/week groups were significantly decreased (*P* < 0.05) relative to those in the UVB group. However, there was no significant difference between the UVB + 150 μg/week group and the UVB + 300 μg/week group (*P* > 0.05) (Fig. 4a, d).

ROS production in the skin tissue was measured in situ dihydroethidium red fluorescence staining (Fig. 4a). UVB exposure significantly increased ROS production (*P* < 0.05), whereas the production of ROS in the UVB + 150 μg/week and UVB + 300 μg/week groups were significantly decreased (*P* < 0.05) in a dose-dependent manner relative to those in the UVB group (Fig. 4e).

### ADSC-EVs increase FB activity and protect FBs from UVB-induced senescence

**Fig. 5:**
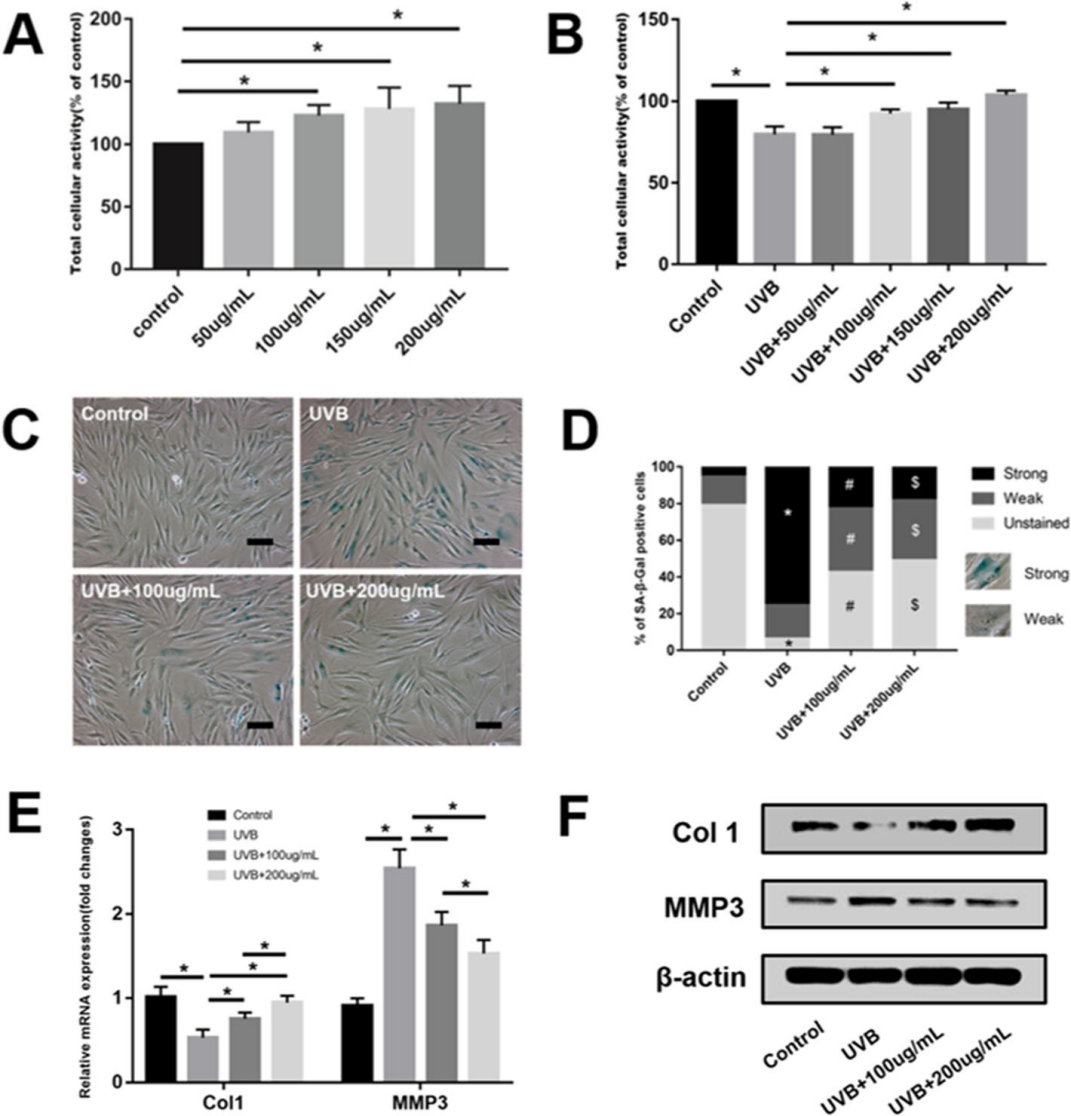
Adipose-derived stem cell extracellular vesicles (ADSC-EVs) increased fibroblast (FB) activity and protected FBs from ultraviolet B (UVB)-induced senescence. Different doses of ADSC-EVs were separately preincubated with FBs (**a**) without or (**b**) with exposure to UVB irradiation. *P < 0.05 indicated a significant difference. (**c**, **d**) Low-dose (100 μg/mL) and high-dose (200 μg/mL) ADSC-EVs ameliorated FB senescence, evaluated by SA-β-Gal staining. Scale bars = 100 μm. *P < 0.05, UVB group compared to the control group. ^**#**^*P* < 0.05 and ^**$**^*P* < 0.05, UVB + 100 μg/mL group, and UVB + 200 μg/mL group compared to the UVB group, respectively. **e**, **f** The mRNA and protein expression of Col-1 and MMP-3 in four groups. *P < 0.05 indicated a significant difference

PBS and 50 μg/mL, 100 μg/mL, 150 μg/mL, and 200 μg/mL EVs were separately preincubated with FBs either exposed or not exposed to UVB irradiation. After 72 h, the CCK-8 results showed that there was a significant dose-dependent increase in cellular activity from 100 μg/mL to 200 μg/mL relative to the PBS controls (*P* < 0.05) (Fig. 5a). Figure 5B shows that UVB significantly decreased cellular activity (*P* < 0.05). However, there was also a significant dose-dependent increase in cellular activity from 100 μg/mL to 200 μg/mL relative to the UVB group (*P* < 0.05). Thus, the low concentration of 100 μg/mL and high concentration of 200 μg/mL EVs were selected to perform subsequent in vitro experiments.

FB senescence was evaluated by SA-β-Gal staining and Col-1 and MMP-3 expression. Compared to the controls, the UVB group showed strong SA-β-Gal staining (*P* < 0.05), whereas the UVB + 100 μg/mL and UVB + 200 μg/mL groups showed significant reductions in SA-β-Gal staining (*P* < 0.05) (Fig. 5c, d). The UVB group showed significantly decreased Col-1 expression and increased MMP-3 expression relative to the control group (*P* < 0.05). The UVB + 100 μg/mL and UVB + 200 μg/mL treatments significantly counteracted the effects of photoaging observed in the UVB group (*P* < 0.05). In addition, the UVB + 200 μg/mL group showed higher Col-1 and lower MMP-3 expression than the UVB + 100 μg/mL group (*P* < 0.05) (Fig. 5e, f).

### ADSC-EVs attenuate raw 264.7 cell differentiation from M0 to M1 macrophages

**Fig. 6:**
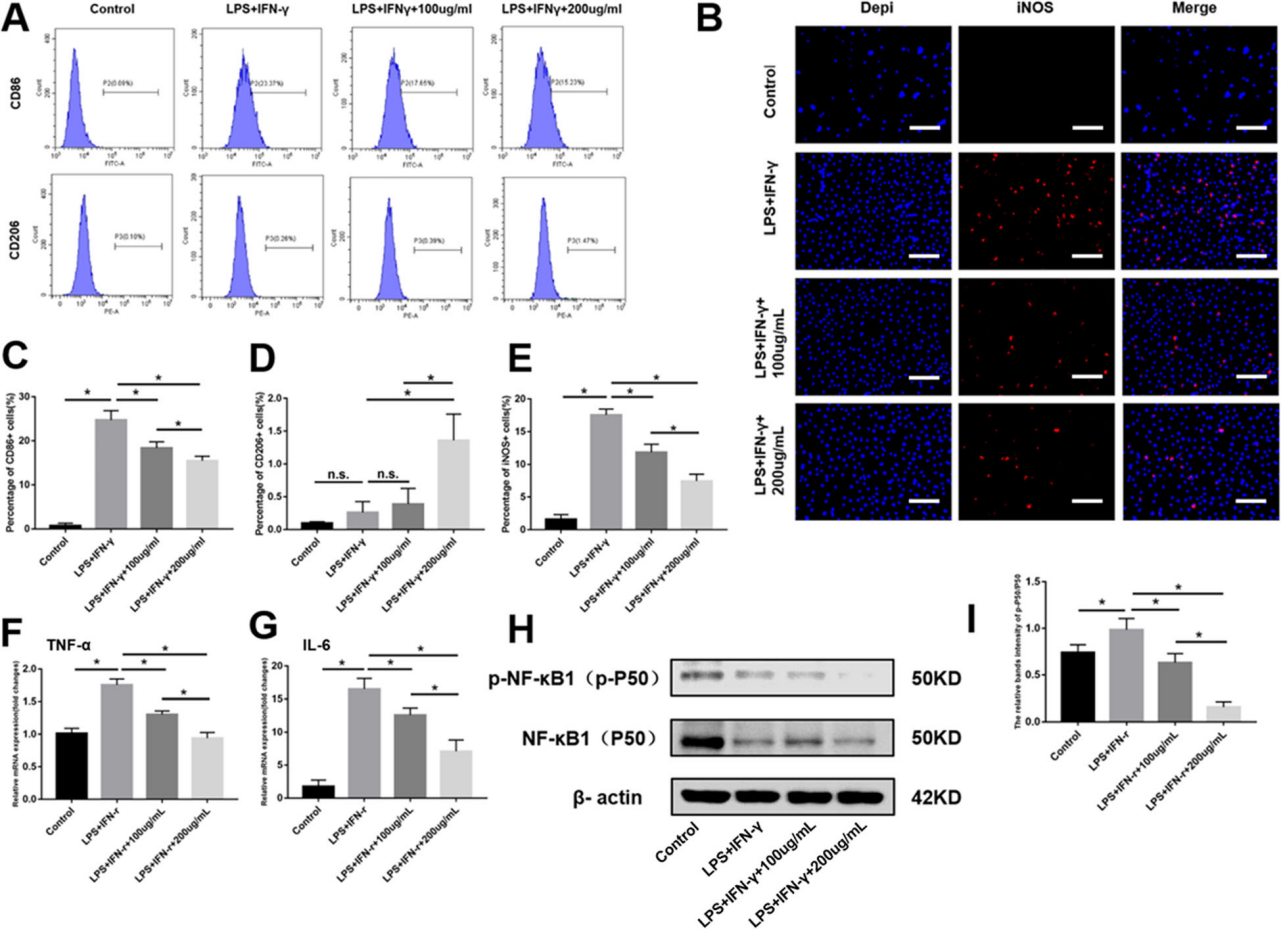
Adipose-derived stem cell extracellular vesicles (ADSC-EVs) attenuated raw 264.7 cell differentiation from M0 to M1 macrophages. **a**, **c**, and **d** After stimulation by lipopolysaccharide (LPS) with IFN-γ, flow cytometry shows that ADSC-EVs significantly attenuated the differentiation of raw 264.7 cells from M0 to M1 macrophages. **b**, **e** Immunofluorescent staining of iNOS and (**f**, **g**) quantitative real-time polymerase chain reaction (qPCR) quantification of TNF-α and IL-6 mRNA expression. **h**, **i** NF-κB1 signaling pathway activation by LPS with IFN-γ, suppressed by ADSC-EVs in a dose-dependent manner. Scale bars = 250 μm. *P < 0.05 indicated a significant difference; n.s., no significant difference between groups

To further confirm the role of ADSC-EVs in attenuating the inflammatory response, raw 264.7 cells were stimulated by LPS with IFN-γ and simultaneously co-incubated with 100 μg/mL or 200 μg/mL EVs. Flow cytometry showed that LPS combined with IFN-γ significantly induced M0 macrophages to differentiate to M1 macrophages (CD86-positive proinflammatory cells), whereas co-incubation with 100 μg/mL or 200 μg/mL EVs significantly attenuated M1 polarization (Fig. 6a, c). Although co-incubation with 200 μg/mL EVs induced increased M2 macrophage (CD206-positive, inhibitory inflammatory cells) polarization relative to other groups, all four groups had low percentages (< 1.5%) of M2 macrophages, indicating that ADSC-EVs did not induce M2 macrophage polarization under these conditions (Fig. 6a, d). Thus, our subsequent experiments focused on the effects of EVs on M1 macrophage polarization.

To further confirm the attenuating inflammatory effect of EVs, immunofluorescence staining, qPCR, and the NF-κB signaling pathway were utilized. Immunofluorescence staining showed that LPS combined with IFN-γ induced significantly more iNOS-positive cells than the control group (*P* < 0.05). After co-incubation with 100 μg/mL or 200 μg/mL EVs, iNOS-positive cells were significantly decreased compared with the LPS + IFN-γ group (*P* < 0.05) in a dose-dependent manner (Fig. 6b, e). Data from qPCR showed that TNF-α and IL-6 (proinflammatory factors) mRNA expression were significantly increased in the LPS + IFN-γ group (*P* < 0.05), whereas after co-incubation with 100 μg/mL or 200 μg/mL EVs, these two proinflammatory factors were significantly decreased in a dose-dependent manner (*P* < 0.05) (Fig. 6f, g). Western blotting showed that p-NF-κB1(p-P50)/NF-κB1(P50) in the LPS + IFN-γ group was significantly increased compared to the controls (*P* < 0.05), indicating that the NF-κB signaling pathway was activated. However, after co-incubation with 100 μg/mL or 200 μg/mL EVs, the p-NF-κB1(p-P50)/NF-κB1(P50) ratio decreased in a dose-dependent manner, indicating that EVs inhibited activation of the NF-κB signaling pathway (Fig. 6h, i).

### ADSC-EVs counteract intracellular ROS production and promote antioxidant enzyme expression

**Fig. 7:**
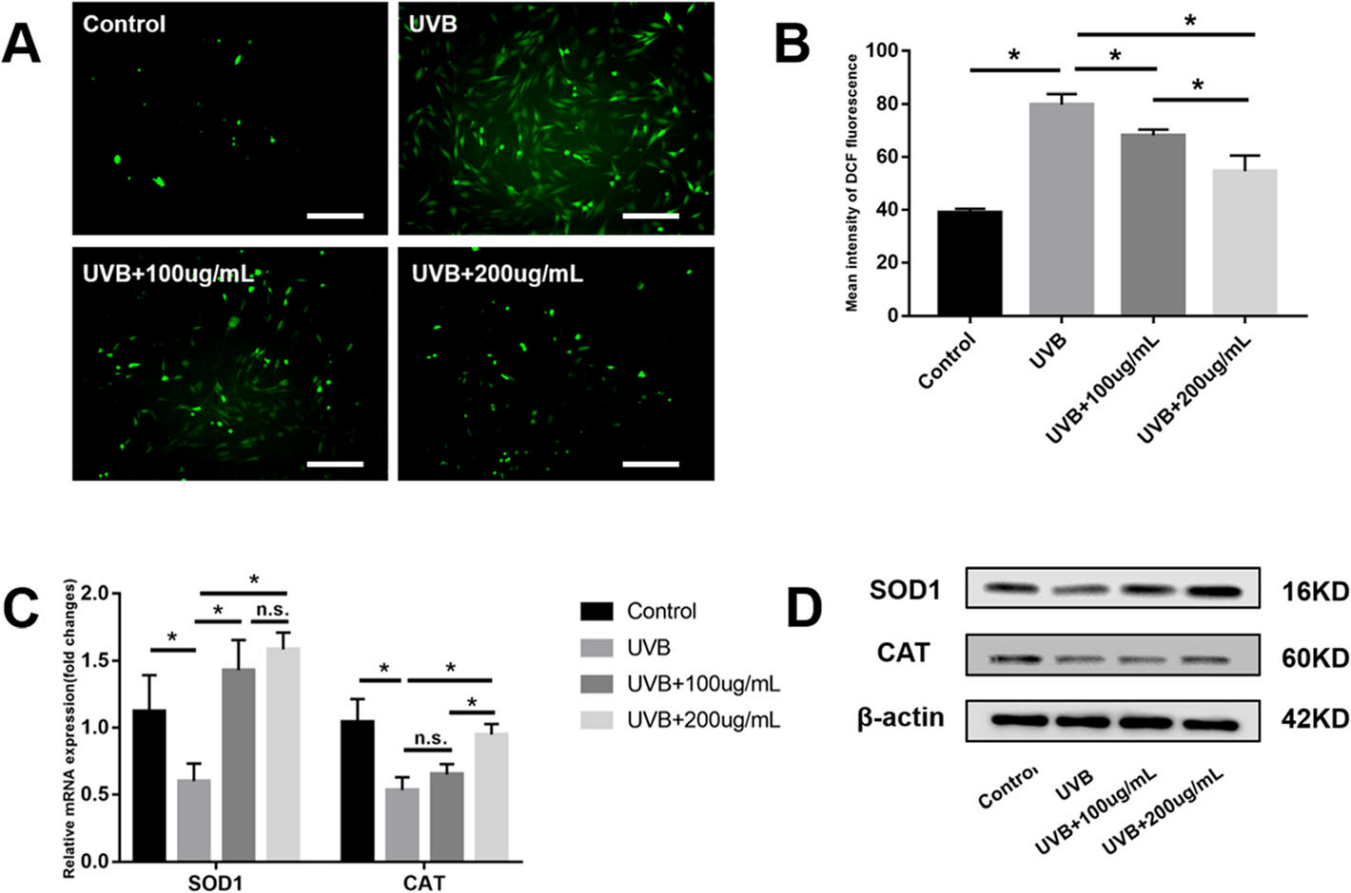
Adipose-derived stem cell extracellular vesicles (ADSC-EVs) counteracted intracellular reactive oxygen species (ROS) production and promoted expression of antioxidant enzymes. **a**, **b** Significantly increased intracellular ROS production by fibroblasts after ultraviolet B (UVB) irradiation, counteracted by ADSC-EVs in a dose-dependent manner. **c**, **d** Antioxidant enzyme (SOD-1 and CAT) expression was decreased after UVB irradiation and increased by ADSC-EVs. Scale bars = 250 μm. *P < 0.05 indicated a significant difference; n.s., no significant difference between groups

To illuminate the mechanism by which ADSC-EVs protect against skin photoaging, ROS production and antioxidant enzyme expression were analyzed. The mean intensity of DCF fluorescence (representing ROS quantity) was significantly increased in the UVB group compared to the control group (*P* < 0.05). However, this intensity was significantly decreased in the UVB + 100 μg/mL and UVB + 200 μg/mL groups compared to the UVB group (*P* < 0.05). In addition, the UVB + 200 μg/mL group showed less ROS production than the UVB + 100 μg/mL group (*P* < 0.05) (Fig. 7a, b).

After UVB irradiation, both mRNA and protein expression of SOD-1 and CAT were significantly decreased (*P* < 0.05). SOD-1 expression in the UVB + 100 μg/mL and UVB + 200 μg/mL groups were significantly increased compared to that in the UVB group (*P* < 0.05), but there was no significant difference between the UVB + 100 μg/mL and UVB + 200 μg/mL groups (*P* > 0.05). CAT expression in the UVB + 200 μg/mL group was significantly increased compared to that of the UVB and UVB + 100 μg/mL groups (*P* < 0.05). There was no significant difference between the UVB + 100 μg/mL group and the UVB group (*P* > 0.05) (Fig. 7c, d).

### ADSC-EVs rescued FB cell cycle arrest

**Fig. 8:**
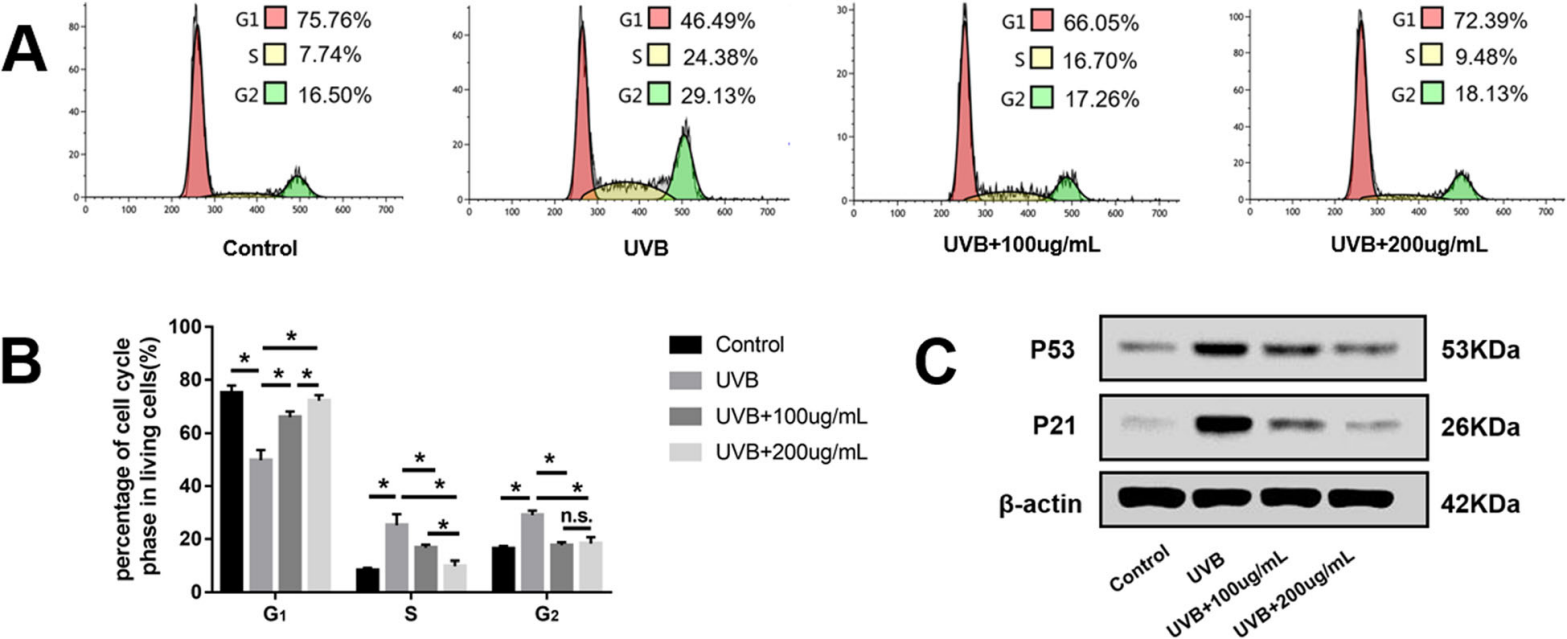
Adipose-derived stem cell extracellular vesicles (ADSC-EVs) rescued fibroblast (FB) cell cycle arrest. **a**, **b** Flow cytometry showing that cell populations in the G1 phases were significantly decreased, whereas those in the S and G2 phases were significantly increased by ultraviolet B (UVB) irradiation. These changes were restored to levels close to those of the control group. **c**, **d** Expression of cell cycle arrest-associated proteins P53 and P21 were increased by UVB irradiation and decreased by ADSC-EVs. *P < 0.05 indicated a significant difference; n.s., no significant difference between groups

Flow cytometry showed that cell populations in the S and G2 phases were significantly increased by UVB irradiation. The UVB + 100 μg/mL and UVB + 200 μg/mL groups showed increased cell cycle arrest, returning to levels close to that of the control group (Fig. 8a, b).

Cell cycle arrest-associated proteins P53 and P21 were also analyzed by western blotting. As shown in Fig. 8c, P53 and P21 protein expression were increased by UVB irradiation. However, they were decreased in the UVB + 100 μg/mL and UVB + 200 μg/mL groups relative to the UVB group, consistent with rescue from FB cell cycle arrest.

## Discussion

We have shown that ADSC-EVs ameliorate UVB-induced skin photoaging in vivo and in vitro in a dose-dependent manner. To explain the mechanism by which ADSC-EVs contribute to anti-photoaging, in vitro studies demonstrated that EVs improved FB activity, counteracted ROS production and cell cycle arrest of FBs, increased Col-1 and antioxidant production, decreased MMP-3 production, and attenuated the inflammatory response of raw 264.7 macrophages.

Previous reports have demonstrated that human induced pluripotent stem cell (iPSC)-derived exosomes ameliorate UVB-induced skin FB photoaging in vitro, and human skin FB-derived exosomes ameliorate UVB-induced mice skin photoaging in vivo [24, 25]. However, ADSC-EVs showed more credible and reliable clinical applications. This is because the liposuction process is highly safe, does not have ethical concerns and allows access to abundant ADSCs [26]. Gentile P et al. showed that ADSCs or ADSCs combined with platelet-rich plasma significantly enhanced fat graft survival rate in a long-term follow-up, indicating a safe and reliable clinical application of ADSCs [27, 28]. ADSCs were also conveniently and reliably used to treat scars and regenerate hair [29, 30]. In contrast, iPSCs are generated by introducing oncogenes such as c-MYC and KLF4 [31]. The application of iPSC-derived exosomes still presents safety concerns. The low quality of the skin from which FBs are generated also limits the application of FB-derived exosomes. In addition to treating skin photoaging, we previously showed that ADSC-EVs combined with hyaluronic acid has a remarkable effect on skin wound healing through promoting fibroblast activity, re-epithelialization, and vascularization [32]. In this regard, ADSC-EVs have composite application potential with other biomaterials.

Although UVA and UVB work together to cause skin photoaging, UVB has a greater contribution owing to its stronger permeability and damaging effects [33, 34]. We therefore used in vitro and in vivo UVB-induced skin photoaging models to evaluate the anti-photoaging effect of ADSC-EVs, which have been widely used to research skin photoaging [35–37].

In this study, ADSC-EVs were successfully isolated and observed internalizing into FBs (Fig. 1), consistent with previous studies [38]. ADSC-EV injections obviously decreased skin wrinkling (Fig. 2), consistent with histological observations (Fig. 3). The greater improvement of the UVB + 300 μg/week group mice compared to those in the UVB + 150 μg/week group may be attributed to a decrease in epidermal thickness and skin β-galactosidase expression, as we observed no difference in dermal thickness (Fig. 3c). The decreased epidermal proliferation index of the UVB group may account for the thickening of the epidermis. This is because the epidermal renewal capacity was weakened, leading to excessive keratinization. Meanwhile, ADSC-EVs increased epidermal cell proliferation and decreased epidermal thickness. The lack of difference in the dermal proliferation index among the four groups (Fig. 4c) suggests that ADSC-EVs may increase dermal thickness by promoting collagen synthesis instead of dermis cell proliferation. The promoting effect of ADSC-EVs on Col-1 synthesis was confirmed via in vitro experiments. MMP-3, which played a role in degrading collagen, was simultaneously suppressed (Fig. 5e, f).

Inflammation is related to skin photoaging [39, 40], and macrophage infiltration plays an important role in the inflammatory response. Staining was therefore performed for the macrophage marker CD68. UVB clearly induced macrophage infiltration, whereas ADSC-EV injection decreased macrophage infiltration (Fig. 4a, d). In vitro results demonstrated that ADSC-EVs decreased raw 264.7 M0 macrophage polarization to M1 after stimulation by proinflammatory factors. However, ADSC-EVs did not increase M0 macrophage polarization to M2 (Fig. 6a, c, d). We therefore focused on the effects of ADSC-EVs on M1 polarization via subsequent in vitro experiments. Immunofluorescence staining combined with qPCR confirmed that ADSC-EVs decreased the polarization of M0 macrophages to M1 (Fig. 6b, e, f, g), suggesting that ADSC-EVs attenuated the inflammatory response in vitro. As a member of the NF-κB family, NF-κB1(P50) is highly expressed at inflammation sites and regulates proliferation, activation, and cytokine production [41]. The increased p-NF-κB1(p-P50)/NF-κB1(P50) ratio in the LPS + IFN-γ group represented activation of the NF-κB1 signaling pathway, whereas ADSC-EVs suppressed NF-κB1 signaling pathway activation in a dose-dependent manner (Fig. 6h, i).

According to previous reports, skin aging as a result of ultraviolet irradiation can be attributed to the generation of ROS that stimulates the inflammatory process [39, 42, 43]. This can be counteracted with antioxidant enzymes [44]. We therefore evaluated ROS production in vivo and in vitro and counteracted with ROS-related antioxidant enzymes in vitro. ADSC-EV treatment counteracted ROS production by increasing the expression of the antioxidant enzymes SOD-1 and CAT (Fig. 7), consistent with previous reports [35, 45].

ROS accumulation has been reported to induce cell cycle arrest [22, 35]. After UVB irradiation, the S and G2 phases of the FB cell cycle were arrested (Fig. 8a, b). The expression of cell cycle arrest-related proteins P53 and P21 were increased (Fig. 8c), similar to results obtained in previous studies [35]. Preincubation with ADSC-EVs prevented cell cycle arrest and decreased P53 and P21 protein expression.

Inflammation and ROS accumulation have been reported to separately activate MMP expression [46–48]. However, as noted above, ROS stimulated the inflammatory process in the skin, whereas activated inflammatory cells such as macrophages produced large quantities of ROS. This indicates that inflammation and ROS accumulation may interact with each other to aggravate MMP activation. MMP activation further degraded skin collagen and resulted in skin photoaging.

ADSC-EVs contain many non-coding RNAs and growth factors, among which miRNAs play an important role in regulating receptor cells [49]. The exact functional miRNAs and their functions in improving skin photoaging will be studied in the future. The unclear primary composition concerning improving UVB-induced skin photoaging was a limitation to this study.

## Conclusions

The anti-photoaging effect of ADSC-EVs was attributed to its attenuation of ROS production and the inflammatory response, two key factors in MMP activation and collagen degradation.

## Supplementary information

**Additional file 1: Supplementary Figure 1**. Characterization of ADSCs. (A) Adipogenic differentiation of ADSCs. Scale bars = 50 μm. (B) Osteogenic differentiation of ADSCs. Scale bars = 50 μm. (C) Chondrogenic differentiation of ADSCs. Scale bars = 50 μm. (D) Surface markers expression of ADSCs.

## Data Availability

The datasets generated during and/or analyzed during the current study are available from the corresponding author on reasonable request.

## Abbreviations

ADSCs: Adipose-derived stem cells
EVs: Extracellular vesicles
UVB: Ultraviolet B
ADSC-EVs: Adipose-derived stem cell extracellular vesicles
H&E: Hematoxylin and eosin
FBs: Fibroblasts
SA-β-Gal: β-galactosidase
Col-1: Collagen 1
MMP-3: Matrix metalloproteinase 3
ROS: Reactive oxygen species
DMEM: Dulbecco’s modified Eagle’s medium
PBS: Phosphate-buffered saline
NTA: Nanoparticle tracking analysis
TEM: Transmission electron microscopy
MED: Minimal erythema dose
DAB: 3,3′-Diaminobenzidine solution
CCK8: Cell counting Kit-8
PMSF: Phenylmethanesulfonyl fluoride
SOD-1: Superoxide dismutase 1
CAT: Catalase
