# Supplementary figures and images for: Extracellular vesicles from adipose-derived stem cells ameliorate ultraviolet B-induced skin photoaging by attenuating reactive oxygen species production and inflammation
Source: Stem Cell Res Ther. 2020 Jul 1;11:264. doi: 10.1186/s13287-020-01777-6 (PMC7329484; doi:10.1186/s13287-020-01777-6)

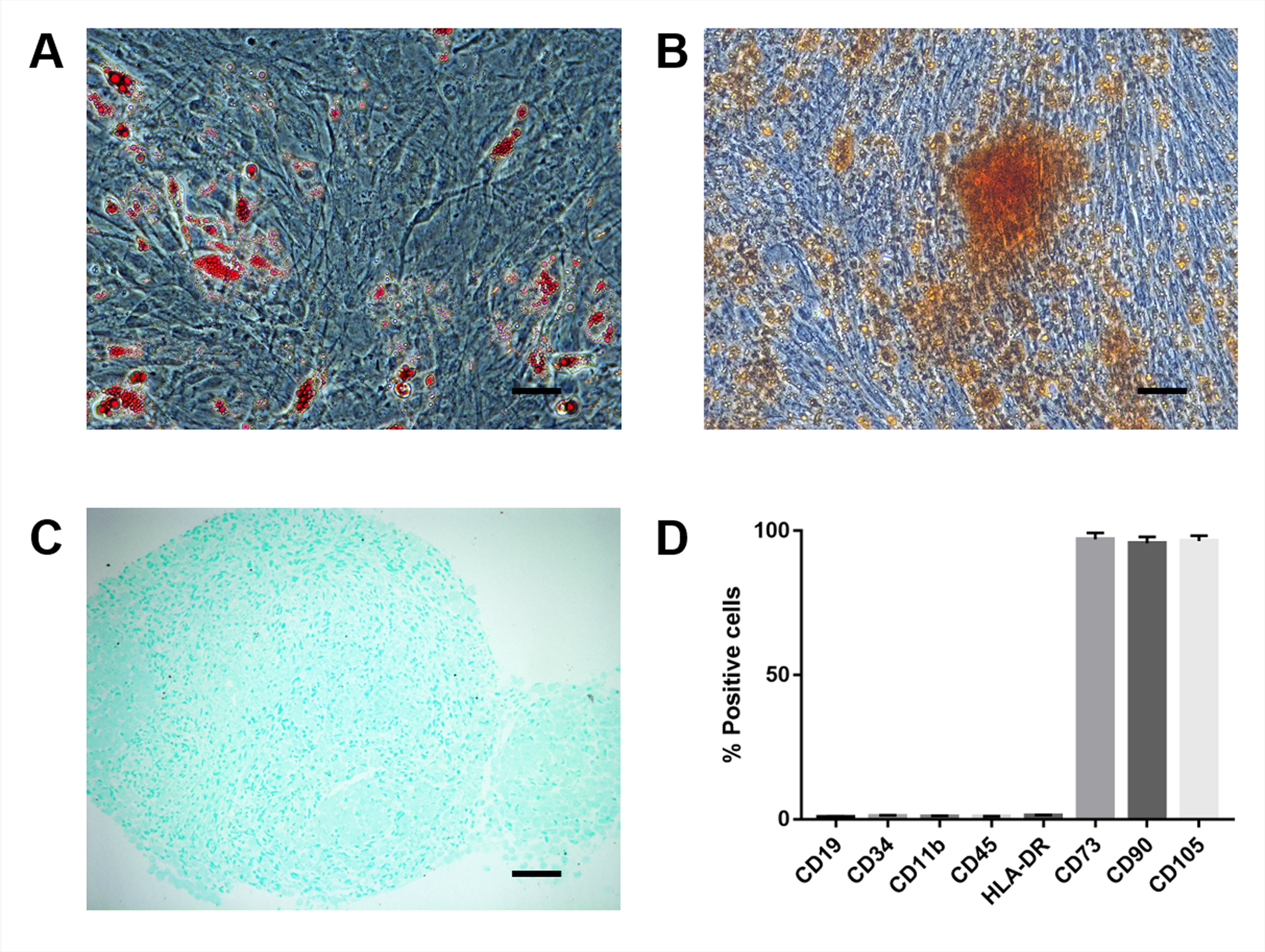

Supplement: Supplementary file 1 — Additional file 1: Supplementary Figure 1. Characterization of ADSCs. (A) Adipogenic differentiation of ADSCs. Scale bars = 50 μm. (B) Osteogenic differentiation of ADSCs. Scale bars = 50 μm. (C) Chondrogenic differentiation of ADSCs. Scale bars = 50 μm. (D) Surface markers expression of ADSCs. [file 13287_2020_1777_MOESM1_ESM.tif]
